# Iron at the Centre of *Candida albicans* Interactions

**DOI:** 10.3389/fcimb.2018.00185

**Published:** 2018-06-05

**Authors:** Ruan Fourie, Oluwasegun O. Kuloyo, Bonang M. Mochochoko, Jacobus Albertyn, Carolina H. Pohl

**Affiliations:** Pathogenic Yeast Research Group, Department of Microbial, Biochemical and Food Biotechnology, University of the Free State, Bloemfontein, South Africa

**Keywords:** *Candida albicans*, host, interaction, iron, polymicrobial, regulation, treatment

## Abstract

Iron is an absolute requirement for both the host and most pathogens alike and is needed for normal cellular growth. The acquisition of iron by biological systems is regulated to circumvent toxicity of iron overload, as well as the growth deficits imposed by iron deficiency. In addition, hosts, such as humans, need to limit the availability of iron to pathogens. However, opportunistic pathogens such as *Candida albicans* are able to adapt to extremes of iron availability, such as the iron replete environment of the gastrointestinal tract and iron deficiency during systemic infection. *C. albicans* has developed a complex and effective regulatory circuit for iron acquisition and storage to circumvent iron limitation within the human host. As *C. albicans* can form complex interactions with both commensal and pathogenic co-inhabitants, it can be speculated that iron may play an important role in these interactions. In this review, we highlight host iron regulation as well as regulation of iron homeostasis in *C. albicans*. In addition, the review argues for the need for further research into the role of iron in polymicrobial interactions. Lastly, the role of iron in treatment of *C. albicans* infection is discussed.

## Introduction

The polymorphic yeast, *Candida albicans*, is one of the organisms which makes up the human microbiome. Although the yeast is largely commensal, it has the potential to become a deadly opportunistic pathogen, especially to individuals with immune deficiency due to organ transplant, chemotherapy or the presence of HIV (Pfaller and Diekema, 2007; Sudbery, 2011). The virulence of *C. albicans* makes it the most common invasive fungal pathogen in humans after *Cryptococcus* and the fourth leading cause of nosocomial bloodstream infections (Brown et al., 2012). Virulence factors associated with *C. albicans* pathogenicity and survival include morphological transition, expression of adhesins, thigmotropism, production of tissue damaging hydrolytic enzymes, and formation of biofilms (Mayer et al., 2013; Sardi et al., 2013). However, for *C. albicans* to express its virulence and pathogenesis within the host, it requires iron (Ramanan and Wang, 2000).

Iron is essential to the survival of most organisms, especially as a cofactor for important metabolic processes, except for the Lyme disease pathogen *Borrelia burgdorferi* which does not require iron but uses manganese instead (Posey and Gherardini, 2000). Lactic acid bacteria have also been reported not to require iron for growth (Archibald, 1983; Pandey et al., 1994), however, according to Duhutrel et al. (2010), in the presence of hematin, myoglobin, and hemoglobin, *Lactobacillus sakei* exhibits enhanced survival in the stationary phase. Additionally, the presence of iron was detected within the cytoplasm of *L. sakei* when grown in the presence of hematin, myoglobin, and hemoglobin (Duhutrel et al., 2010). This observation indicates that certain lactic acid bacteria can take up iron and may even utilize it differently.

The important roles assigned to iron proteins include the transportation and activation of molecular oxygen, reduction of ribonucleotides, and dinitrogen, and the activation and decomposition of peroxides (Pierre et al., 2002). Although iron is abundant within the earth crust, in the human host, iron is a highly restricted nutrient (Frey and Reed, 2012; Hood and Skaar, 2012). The need for iron restriction is due to its propensity to catalyze the formation of reactive free oxygen radical species with hydrogen or lipid peroxide, which have a damaging effect on cellular components (Philpott, 2006). Furthermore, its demand by pathogenic microbes necessitates the host to restrict its availability as a defense mechanism known as nutritional immunity (Weinberg, 1975). Therefore, *C. albicans* has developed sophisticated systems to scavenge iron within the host for survival (Eck et al., 1999; Ramanan and Wang, 2000; Weissman et al., 2002). This review aims to discuss iron as a vital component of the human host, as well as how *C. albicans* can exploit host-derived iron. In addition, focus is placed on the regulation of iron acquisition in this yeast. This review also discusses the known roles of iron in *C. albicans*-polymicrobial interactions, as well as manipulation of iron availability as treatment options for *C. albicans* infection.

## Iron in living systems

Iron is a vital micronutrient for all living organisms. It plays a central role in biological processes and its availability is essential for biogenesis of cellular components and metabolism (Crichton and Pierre, 2001). Iron is found in numerous proteins, including in haem groups and iron-sulfur clusters (Beinert et al., 1997). These proteins are involved in iron transport and storage, DNA synthesis and mitochondrial respiration (Beinert et al., 1997; Lill, 2009). The two oxidation states of iron, ferrous (Fe^2+^) and ferric (Fe^3+^), give this metal a chemical character that allows for its involvement in important enzymatic reactions where electron transfer is essential (Philpott, 2006). The oxidized form of iron, ferric (Fe^3+^) form, is present in aerobic environments, where it reacts with oxygen to form oxides and oxyhydroxide polymers that have a relatively low solubility (10^−9^ M) at neutral pH (Aisen et al., 2001). The more soluble reduced form, ferrous (Fe^2+^) iron, reacts with oxygen to form toxic reactive oxygen species (ROS) through Fenton reactions (Winterbourn, 1995; Ilbert and Bonnefoy, 2013). Reactive oxygen species (ROS) are highly reactive molecules and can oxidize cell components such as lipids, proteins, or DNA thereby disrupting cellular integrity (Scherz-Shouval and Elazar, 2007). Both pathogens and the host have developed precise mechanisms that regulate iron uptake, utilization, and storage to prevent the threat posed by ROS (Barber and Elde, 2015).

## Host iron homeostasis

### Iron distribution within the host

Iron in the human host is largely unavailable as it is constantly sequestered by specialized proteins which prevent attack by ROS or exploitation by pathogens (Ganz and Nemeth, 2015). An average adult human body contains approximately 3−5 g of iron (approximately 44−55 mg.kg^−1^ body weight), of which 66% circulates within the blood as a component of hemoglobin within the erythrocytes (Mendel, 1964; Caza and Kronstad, 2013). Haemoglobin is a protein comprised of two alpha-beta pairs of globin chains each encircling an iron-tetrapyrrole ring called haem (Marengo-Rowe, 2006). The haem group contains iron as a co-factor at its core and the iron reversibly binds oxygen only when it is in the ferrous form (Fe^2+^) (Marengo-Rowe, 2006). The remaining iron within the host is either coupled to ferritin within the hepatocytes (~1,000 mg); associated with macrophages in spleen, liver, and bone marrow (~600 mg) or attached to smaller haem proteins such as myoglobin (~300 mg) (Mendel, 1964; Andrews, 1999). A smaller portion of body iron functions as a co-factor in enzymes (e.g., cyclooxygenases and ribonuclear reductases) or is found attached to iron-binding proteins such as transferrin and lactoferrin (Lambert, 2012; Tandara and Salamunic, 2012). Daily iron uptake from diet via duodenal enterocytes is minimal (1–2 mg per day) compared to daily iron requirements, especially for hemoglobin synthesis, which requires approximately 20−25 mg iron (Ganz and Nemeth, 2012). Thus, iron is frequently recycled from damaged or senescent erythrocytes (lifespan 120 days) by macrophages via erythrophagocytosis (erythrocyte ingestion by macrophages) to supply close to 95% of daily iron requirements (Johnson and Wessling-Resnick, 2012). Haem-bound iron is released from erythrocytes within macrophages by haem oxygenase (HO-1) and is further transported to the bone marrow for erythropoiesis via transferrin (Ganz and Nemeth, 2012). Haem oxygenase 1 (HO-1) proteolytically degrades haem to yield carbon monoxide, biliverdin, and ferrous iron (Fe^2+^), which is shuttled to the extracellular fluids by iron exporter ferroportin, present on the cell surfaces of macrophages (Colas and Ortiz De Montellano, 2003; Santos et al., 2003; Pendrak et al., 2004). Several molecules, including haptoglobin, haemopexin, albumin, and both high- and low-density lipoproteins (HDL and LDL) bind haem that is released into the bloodstream during haemolysis, in order to reduce iron toxicity and increase efficiency of iron recycling (Ascenzi et al., 2005; Caza and Kronstad, 2013). This is essential in limiting iron availability to invading pathogens, as both bacterial and fungal pathogens have been shown to target haem, hemoglobin, and other iron-complex proteins (Ganz and Nemeth, 2015).

### Iron regulation within the host

Systemic iron levels are tightly regulated by iron metabolism master regulator, hepcidin (encoded by *HAMP*), while individual cells may independently regulate iron by receptor-mediated endocytosis of transferrin (Ganz and Nemeth, 2015). Hepcidin is a liver-derived hormone that regulates introduction of iron into plasma from three major sources: iron stored within hepatocytes, absorbed iron in the duodenal enterocytes and iron recycled from macrophages (Ganz and Nemeth, 2012; Pantopoulos et al., 2012). Hepcidin binds to ferroportin—an iron-exporting protein expressed on the plasma membrane of macrophages, hepatocytes, and enterocytes—resulting in ferroportin endocytosis and degradation, thereby greatly reducing iron export to the extracellular fluids (Finberg, 2013). Elevated concentrations of hepcidin increase iron sequestration by macrophages and decrease duodenal iron absorption during inflammatory reactions and thus reduces plasma iron levels (Cassat and Skaar, 2013; Noble, 2013). Hepcidin has been reported to be induced by high levels of dietary iron, inflammation, and bacterial lipopolysaccharide (LPS) (Haley and Skaar, 2012). During periods of high iron requirement, such as in iron deficiency or pregnancy, its levels are reduced to increase duodenal iron absorption and release from macrophages (Anderson and Vulpe, 2009). Deletion of the gene associated with hepcidin (*HFE*-High iron gene) in mice, resulted in excessive iron overload, i.e., haemochromatosis, a condition characterized by high iron levels, presence of free oxygen radicals and cell damage (Ganz, 2003; Schaible and Kaufmann, 2004). The *HFE* gene encodes for a major histocompatibility complex (MHC) class I molecule, HFE, which associates with TfR1 (Transferrin receptor 1) to mediate iron uptake especially in the duodenum (Pantopoulos, 2008). Mutations in the HFE gene in humans result in high iron uptake and hepcidin deficiency, leading to iron overload (Ganz, 2003). Retained high levels of this hormone within the plasma have been associated with hypoferremia and iron deficiency anemia (Kim et al., 2014).

### Host defence and nutritional immunity

Iron homeostasis and bioavailability to either the host or invading pathogens is tightly regulated through coordination of hepcidin, iron binding proteins and several enzymes involved in iron metabolism (McKie et al., 2001; Nemeth et al., 2004). The process of sequestering iron by the host and restricting its bioavailability to invading pathogens is called nutritional immunity (Weinberg, 1974). In the circulatory system, extracellular iron transport to various cell types is facilitated by transferrin, a plasma protein that has a high affinity (*K*_d_ ~10^−22^ M) for iron (Aisen and Brown, 1977). However, only 30–40% of transferrin is saturated with iron, as circulating transferrin concentrations (~30 μM) can bind 60 μM of iron, and only 18–21 μM iron concentrations have been reported within the plasma (Weinberg, 1971). This leaves approximately 10^−18^ M free iron, which is inaccessible to bloodstream pathogens, even though transferrin itself may be a target of pathogens for iron exploitation during bloodstream infections such as in candidaemia (Knight et al., 2005; Caza and Kronstad, 2013). Three types of transferrin proteins have been defined: serum transferrin (apotransferrin in iron free-form), secretory lactoferrin—found in extracellular secretions such as milk, (released also by leukocytes); and ovotransferrin which is present in egg-white (Masson et al., 1966; Masson and Heremans, 1971; Han, 2014b). Transferrin transfers iron to cells through transferrin receptor 1 (TfR1) found on the surface of most body cells, and via transferrin receptor 2 (TfR2) expressed mainly in hepatocytes (Anderson and Vulpe, 2009; Caza and Kronstad, 2013). Transferrin-Transferrin receptor (TfR1) interaction triggers cell-mediated endocytosis of the complex and iron bound on transferrin dissociates within endosomes at a low pH (~5.5) since apotransferrin binds iron with a higher affinity at physiological pH relative to a low pH (Bali and Aisen, 1992; Anderson and Vulpe, 2009). During infections, pro-inflammatory cytokines play an essential role in hepcidin-independent sequestration of iron. Tumour necrosis factor alpha (TNF-α), interleukins (IL-1 and IL-6), and interferon gamma (IFN-γ) increase uptake of free iron by macrophages and transferrin through upregulation of transferrin receptor proteins (Byrd and Horwitz, 1993; Ludwiczek et al., 2003; Drakesmith and Prentice, 2012).

Since most of the iron within the host circulates in the blood bound to hemoglobin within erythrocytes, several mechanisms are deployed by the host to quickly remove haem and hemoglobin during haemolysis or dietary haem absorption (Haley and Skaar, 2012). Haptoglobin and haemopexin rapidly bind hemoglobin and haem released from erythrocytes respectively for iron recycling and to possible avoid toxicity posed by free haem (Kumar and Bandyopadhyay, 2005; Hood and Skaar, 2012). Haemopexin-bound haem is transported to the liver for recycling and liver parenchymal cells take up the complex via receptor-mediated endocytosis (Tolosano et al., 2010). Serum albumin can bind free haem in cases of enhanced haemolysis, although it remains unclear if albumin can transport haem to the liver (Tolosano et al., 2010). Some extracellular and membrane-associated proteins essential in sequestering iron within the host, especially during microbial infections and immune response, have also been described (Ganz and Nemeth, 2015). These include siderocalin [also referred to lipocalin-2 or neutrophil gelatinase-associated lipocalin (NGAL)] and the natural resistant-associated macrophage protein 1 (NRAMP1) (Forbes and Gros, 2001; Chakraborty et al., 2012). Siderocalin is produced mainly by neutrophils, macrophages, and epithelial cells through cytokine-mediated response, especially IL-1β (Borregaard and Cowland, 2006). Siderocalin binds to several kinds of siderophores including bacterial enterobactin (*Escherichia coli, Klebsiella*, or *Salmonella* spp) and mycobactin (*Mycobacterium tuberculosis*), thereby subverting the pathogen's access to siderophore-complexed iron (Flo et al., 2004; Johnson and Wessling-Resnick, 2012). On the other hand, NRAMP1 is a pH dependent phagosomal protein, associated with macrophages and neutrophils (Vidal et al., 1995, 1996). This protein is a divalent metal ion transporter that pumps iron out of the phagolysosome into the macrophage cytoplasm to limit iron-access to intracellular pathogens (Kuhn et al., 2001).

As the above information reflects, iron homeostasis is tightly regulated for proper cellular functioning as well as for immune response. Global iron homeostasis is disturbed during systemic candidiasis. In a murine model, macrophages were shown to aggregate around fungal lesions, concomitantly reducing iron availability for the fungus (Potrykus et al., 2013). In addition, hepcidin release is increased during candidiasis, inhibiting release of iron from stores. This in turn leads *C. albicans* to alter its iron acquisition strategy as disease progresses. Interestingly, *C. albicans* has been shown to increase hepcidin production possibly via the signal transducer and activator of transcription 3 (STAT3) pathways, which also plays a role in T helper cell 17 (Th17) inflammatory responses (Armitage et al., 2011; Drakesmith and Prentice, 2012). T helper cell 17 (Th17) is critical in *C. albicans* host-clearance during inflammatory response (Hernandez-Santos and Gaffen, 2012). Moreover, the study of Armitage et al. (2011) demonstrated that *C. albicans* reduced transferrin saturation from ~70 to 15% in infected mice although there was no significant correlation between induced hepcidin and transferrin saturation (Armitage et al., 2011). *C. albicans* has also been shown to utilize transferrin as iron source *in vitro* as demonstrated by Knight and co-workers, though transferrin is generally known to inhibit *C. albicans* growth (Caroline et al., 1964; Esterly et al., 1967; Han, 2005; Knight et al., 2005).

## Iron acquisition by *Candida albicans*

In order to obtain iron from the host, *C*. *albicans* may use either a high affinity reductive system, a siderophore uptake system or the hemoglobin–iron uptake system for iron acquisition (Moors et al., 1992; Ramanan and Wang, 2000; Lesuisse et al., 2002). Iron acquisition via the reductive system involves the reduction of ferric iron to the ferrous form with the subsequent transport of the reduced ferrous iron into the cell via a ferrous transporter complex (reviewed in Philpott, 2006). Although *C. albicans* has not been reported to use lactoferrin as an iron source, the reductive system can be used to acquire iron from sources such as ferritin and transferrin as well as free iron (Knight et al., 2005; Almeida et al., 2008). In *C. albicans*, two ferric reductases, Cfl1 (Fre1) (Yamada-Okabe et al., 1996; Hammacott et al., 2000) and Cfl95 (Fre10, Rbt2) (Knight et al., 2002), have been characterized. Within the *C*. *albicans* genome, there are as much as 17 ferric reductase-related genes but not all these genes are likely to be a ferric reductase. A functional ferric reductase has a ferric reductase domain and a FAD and/or NAD binding domain. These domains were absent in two of the reported reductases while another reductase was lacking a FAD and NAD binding domain (reviewed by Almeida et al., 2009). During iron acquisition, *C. albicans* ferric reductases Cfl1 and Cfl95, localized in the plasma membrane, reduce ferric iron to a soluble ferrous state (Xu et al., 2014b; Yu et al., 2014). Due to the spontaneous generation of toxic free radicals by ferrous iron, a multicopper oxidase enzyme is involved in the reconversion of the produced ferrous iron to a ferric state; a reaction known as ferroxidation (reviewed by Kosman, 2003; Almeida et al., 2009). The oxidation of iron by multicopper oxidases requires copper, and this is supplied by the intracellular copper transporter Ccc2 (Weissman et al., 2002). Finally, the iron permease, Ftr1, forms a complex with the multicopper oxidases to transport the ferric iron into the cell (Ziegler et al., 2011; Mamouei et al., 2017). Another mechanism of iron acquisition in *C*. *albicans* is via the low molecular weight ferrichrome as well as ferrioxamine-type siderophores which chelate iron with a high affinity (Minnick et al., 1991; Ardon et al., 2001). The analysis of the *C. albicans* genome has not revealed a siderophore synthesis pathway (Lan et al., 2004). In *C. albicans*, there is only one siderophore transporter which is the Sit1/Arn1 transporter. Since *C. albicans* does not directly produce siderophores, it then takes up xenosiderophores using the Sit1/Arn1 transporter or reductive iron uptake pathway (Ardon et al., 2001; Heymann et al., 2002).

**Figure 1:**
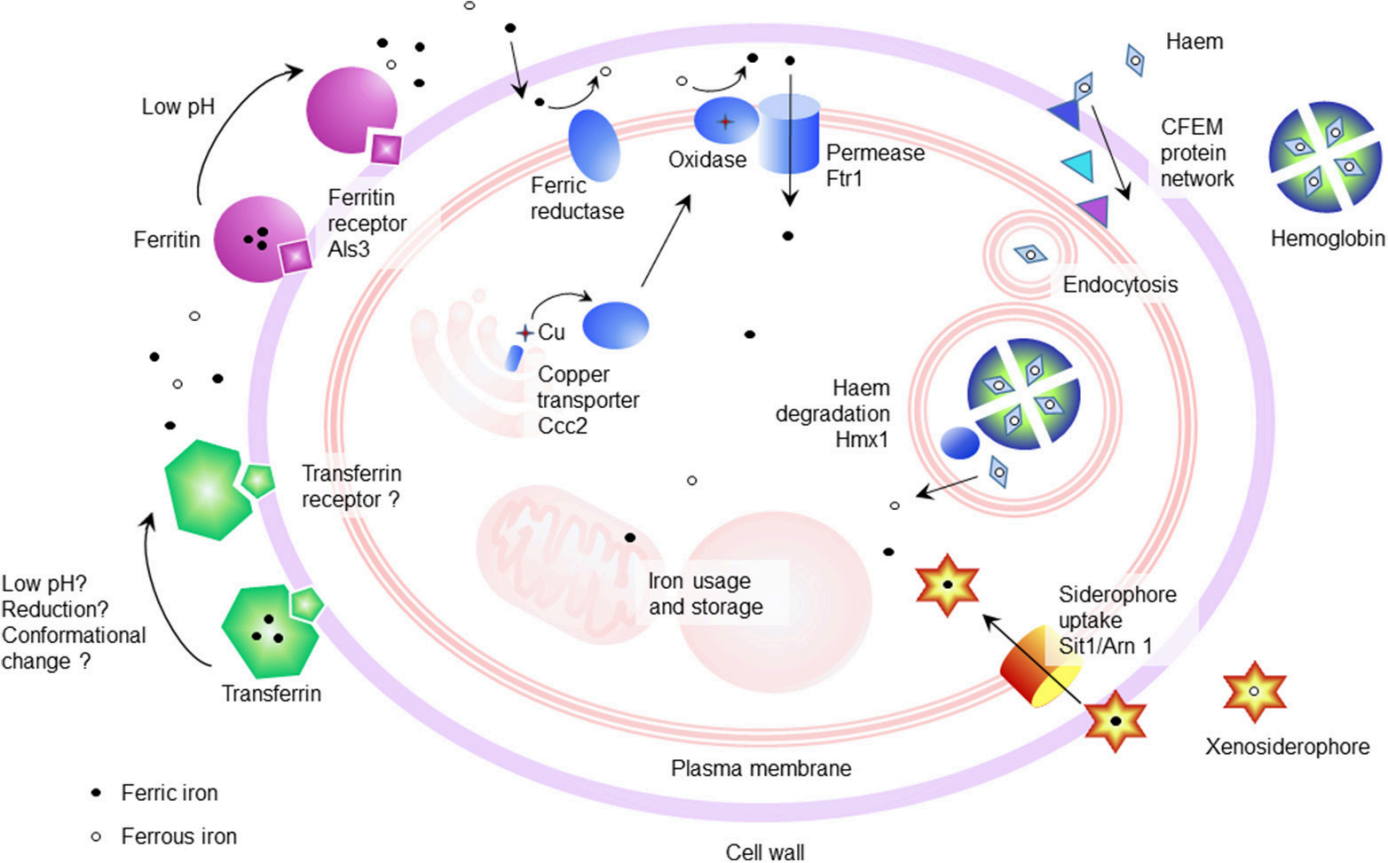
Schematic of the iron uptake mechanisms of *Candida albicans*. Iron can be acquired from host iron binding proteins such as hemoglobin (Kuznets et al., 2014), ferritin (Almeida et al., 2008), and transferrin, as well as from free iron through the action of the reductive iron uptake pathway (Knight et al., 2002, 2005). In addition, the siderophore uptake system (Sit1/Arn1) functions in iron acquisition from xenosiderophores (Heymann et al., 2002).

Haemin and hemoglobin, which make up 70% of the potentially available iron in the host, can also be utilized by *C. albicans* through haemolytic activity (Manns et al., 1994). The uptake of iron from haem proteins does not depend on the high affinity system, but rather relies on the common in several fungal extracellular membrane (CFEM) proteins (Kuznets et al., 2014). The CFEM domain is characterized by eight cysteine residues of conserved spacing and commonly found in many fungi membrane proteins (Kulkarni et al., 2003). In *C. albicans*, CFEM proteins namely Rbt5, Pga10 (Weissman and Kornitzer, 2004; Weissman et al., 2008), Pga7 (Kuznets et al., 2014), and Csa2 (Nasser et al., 2016) are responsible for haem iron uptake. Rbt5, a GPI-anchored haem-receptor, works in combination with Pga7, and possibly the secreted hemophore Csa2, to shuttle haem across the cell wall to the plasma membrane (Kuznets et al., 2014). A schematic representation of the iron uptake mechanisms can be seen in Figure 1.

## Regulation of iron acquisition in *Candida albicans*

**Figure 2:**
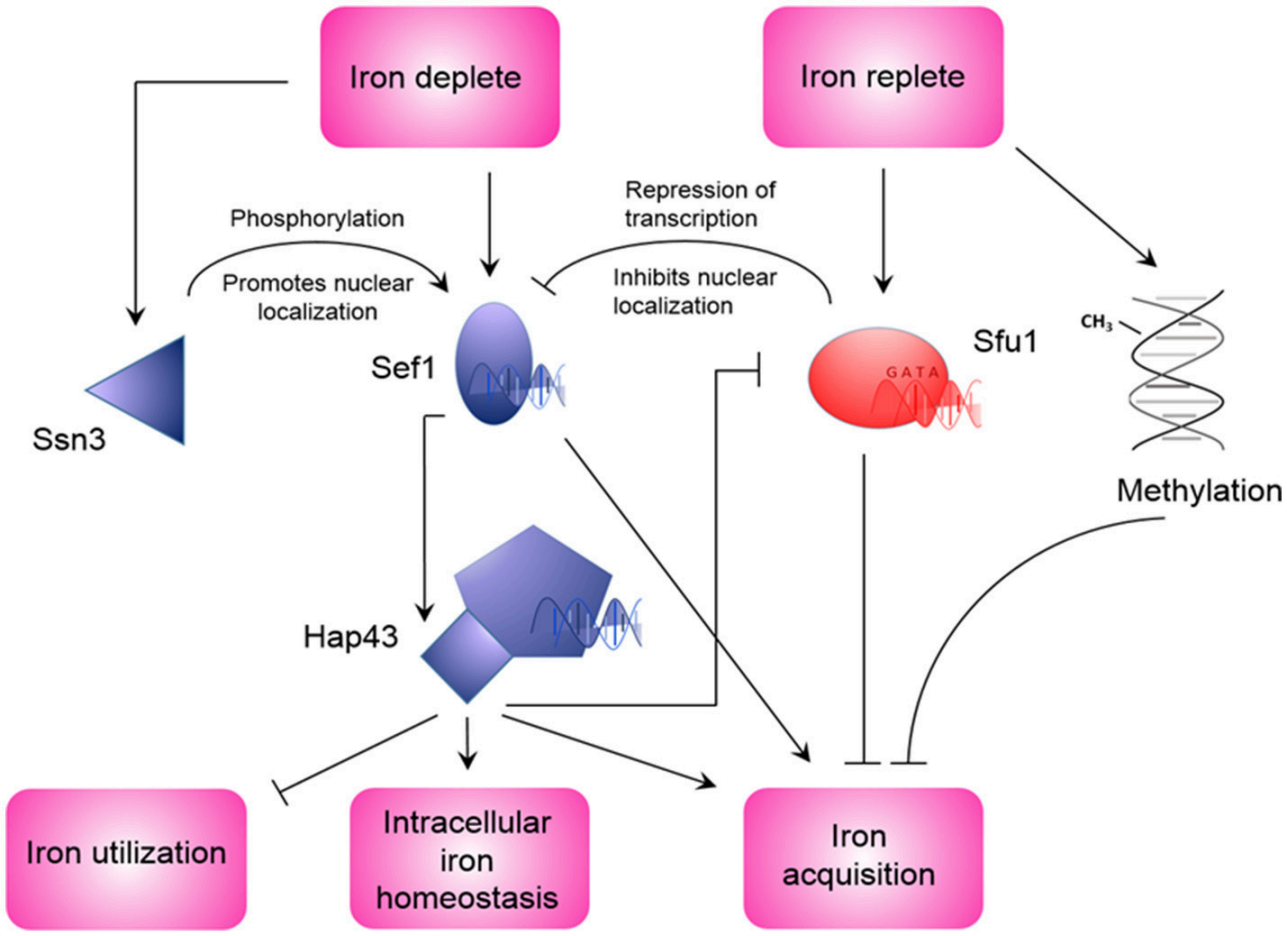
Regulation of iron homeostasis in *Candida albicans*. During iron depleted conditions, Sef1 is induced and activated by Ssn3 (Chen and Noble, 2012), and induces the expression of HAP43 and iron acquisition genes (Chen et al., 2011). In turn, Hap43 also induces iron acquisition genes, as well as repression of iron utilization genes and Sfu1 (Singh et al., 2011). During conditions of replete iron, repression of Sfu1 expression is lifted. This leads to inhibition of Sef1 expression and nuclear localization, as well as repression of iron acquisition genes (Lan et al., 2004). Iron replete conditions can also promote methylation of DNA that can lead to repression of iron acquisition genes (Mishra et al., 2011).

The duality of *C. albicans* as both a commensal and pathogen of humans, exposes it to differences in nutrient availability that are associated with different environments within the human body (Blankenship and Mitchell, 2011). This extends to iron availability. During infection, nutritional immunity, and iron-binding proteins may make iron largely unattainable, however, during commensalism in the oral cavity and gastrointestinal tract, iron may be more freely available. This forces a need on *C. albicans* to regulate iron acquisition to circumvent iron toxicity as well as iron deficiency in environments with different iron availability (Kronstad, 2013). The robustness of iron acquisition is tightly linked to the stage of infection, for example, in the initial stage of infection, gene expression is characterized by a strong iron acquisition strategy due to limited iron availability, whereas during the late infection stage, more iron is available due to extensive tissue damage (Lin C. et al., 2014; Xu et al., 2015). To compensate for changes in iron availability, proteins involved in iron acquisition are differentially expressed (Sorgo et al., 2013). For example, the expression of the ferritin receptor, Als3, increases in the hyphae-associated program, and inhibition of the hyphal form leads to its diminished expression and reduced capability of ferritin-binding (Almeida et al., 2009). During systemic infection with occurrence of *C. albicans* in the bloodstream, low iron availability leads to the expression and nuclear localization of Sef1 (Chen et al., 2011). This Cys_6_Zn_2_ transcription factor induces the expression of iron uptake genes, virulence-associated genes as well as the expression of *HAP43*, the subunit of the CCAAT-binding factor with transcriptional activator function (Baek et al., 2008). Hap43 in turn represses iron consuming processes and induces iron uptake genes (Baek et al., 2008; Singh et al., 2011). Interestingly, the CCAAT-binding factor also plays a role in the regulation of the oxidative stress response in *C. albicans* (Chakravarti et al., 2017). In addition to this, Hap43 represses the GATA family transcription factor Sfu1. This transcription factor prevents toxicity in iron replete medium through inhibition of Sef1, and indirectly Hap43, thereby repressing genes involved in iron acquisition (Lan et al., 2004). This suppression of Sef1 by Sfu1 is not only mediated by transcriptional inhibition, but Sfu1 has also been shown to physically associate with Sef1 and sequester it in the cytoplasm, where it is unable to perform transcriptional activation of iron acquisition genes (Chen and Noble, 2012). In addition to Sfu1, Sef1 is also post-transcriptionally regulated by the protein kinase Ssn3. Under iron-depleted conditions, Ssn3 forms a complex with Sef1 and activates it via phosphorylation. Also, Ssn3 promotes the nuclear localization of Sef1, where Sef1 can induce iron-acquisition genes. This information places Sef1 as a promoter of virulence, through its ability to enable *C. albicans* to live in the iron-deficient environment of the mammalian host, as well as placing Sfu1 as a promoter of commensalism through protecting *C. albicans* from iron-mediated toxicity in the iron-replete environment of the gastrointestinal tract (Chen and Noble, 2012). A summary of the regulation of genes involved in iron uptake and regulation of iron by Sef1, Sfu1, and Hap43 is given in Table 1. This information was obtained through PathoYeastract (Monteiro et al., 2017), a database for regulatory associations between transcription factors and target genes. Table 1 includes documented regulatory associations, where, in the case of iron in *C. albicans*, it is based on the work of Chen et al. (2011), Lan et al. (2004), and Singh et al. (2011), as well as potential regulation based on the presence of putative DNA-binding sites in the promoter region of genes. In addition to the aforementioned transcription factors, transcription of iron acquisition genes such as *RBT5* is also inhibited by methylation during iron-replete conditions (Mishra et al., 2011). Interestingly, these authors also observed an increased frequency of mutation in genes that are repressed under iron-replete conditions. A schematic of *C. albicans* iron regulation can be seen in Figure 2.

**Table 1:** Known regulatory associations between transcription factors and genes associated with iron uptake or homeostasis obtained from PathoYeastract (Monteiro et al., 2017).

**System**	**ORF**	**Gene name**	**Sef1**	**Sfu1**	**Hap43**
Ferric	orf19.1263	*CFL1*			
reductases	orf19.1264	*CFL2*			
	orf19.1270	*FRE3*			
	orf19.1930	*CFL5*			
	orf19.1932	*CFL4*			
	orf19.6138	*CR_07300W_A*			
	orf19.7077	*C7_00430W_A*			
	orf19.701	*CFL11*			
	orf19.3538	*FRE9*			
	orf19.1415	*FRE10*			
	orf19.2312	*C1_11020W_A*			
	orf19.5634	*FRP1*			
	orf19.7112	*FRP2*			
	orf19.1844	*CR_06870C_A*			
	orf19.6139	*FRE7*			
	orf19.867	*C2_03530W_A*			
	orf19.4843	*C1_09780C_A*			
Multicopper	orf19.4211	*FET31*			
oxidases	orf19.4213	*FET3*			
	orf19.943	*FET33*			
	orf19.4215	*FET34*			
	orf19.4212	*FET99*			
	orf19.4328	*CCC2*			
Iron	orf19.7219	*FTR1*			
permeases	orf19.7231	*FTR2*			
	orf19.4802	*FTH1*			
	orf19.3227	*FTH2*			
Ferritin uptake	orf19.1816	*ALS3*			
Siderophore uptake	orf19.2179	*SIT1*			
Haemoglobin/	orf19.5636	*RBT5*			
Haem uptake	orf19.7114	*CSA1*			
	orf19.3117	*CSA2*			
	orf19.5635	*PGA7*			
	orf19.5674	*PGA10*			
	orf19.6073	*HMX1*			
Regulation	orf19.3753	*SEF1*			
	orf19.4869	*SFU1*			
	orf19.681	*HAP43*			
Intracellular iron homeostasis	orf19.2178	*MRS4*			
	orf19.6948	*CCC1*			
	orf19.2069	*SMF3*			

*Dark gray indicates documented regulation whereas light gray indicates potential regulation*.

In addition to the regulation of systems for iron uptake from the extracellular environment, the localization and storage inside the cell is of considerable importance. Dysregulation of intracellular iron homeostasis may lead to cell cycle arrest and autophagy (Dong et al., 2017). Xu et al. (2014a) identified a pathway that controls intracellular iron levels, including mobilization from storage compartments such as vacuoles and mitochondria. This pathway includes the mitochondrial carrier Mrs4 and the two vacuolar transporters Ccc1 and Smf3 with contrasting roles (Ccc1 promotes vacuolar storage of iron, whereas Smf3 plays a role in vacuolar export). Importantly, all three of these genes are targets of regulation by Hap43 (Chen et al., 2011; Singh et al., 2011; Monteiro et al., 2017), linking regulation of iron not only to acquisition from external sources but also intracellular homeostasis.

Interestingly, large scale gene expression and chromatin remodeling was identified to take place in response to iron (Puri et al., 2014). The Hog1 and Cek1 MAPK signaling pathways, known to play a role in stress responses, as well as in adhesion and biofilm formation, were activated in response to high iron (Kaba et al., 2013; Puri et al., 2014). This may indicate that cells respond to iron availability as an indication of their environment, i.e., the iron replete gastrointestinal tract where increased adhesion and resistance to oxidative stress would be beneficial. Indeed, Pande et al. (2013) showed that passage of *C. albicans* through the gastrointestinal tract triggers the epigenetic Gastrointestinally-indUced Transition (GUT) phenotype, where a decrease in iron acquisition is seen in response to the iron-replete environment of the gastrointestinal tract. This serves as evidence that iron availability leads to a fine-tuned response via transcription factors for iron acquisition, as well as chromatin level and genome wide changes where iron availability may act as cues for specific niches requiring different adaptations.

## Role of iron in polymicrobial interaction of *Candida albicans* and bacteria

The microbiome has the ability to alter physiology of the host, including metabolism and immunity (Nash et al., 2017). In addition, members of the microbiome may drastically affect the growth of co-habitants (Hoffmann et al., 2013). Compared to bacteria, fungal constituents can be as low as 0.1% of the total microbiota and may show large diversity (Ghannoum et al., 2010; Qin et al., 2010). The microbiome, including the mycobiome, is largely dependent on the area of the body, and may be altered during disease (Pragman et al., 2010; Mukherjee et al., 2014; Gouba and Drancourt, 2015). For example, *Candida* is more prevalent among HIV infected subjects. In addition, broad spectrum antibiotic treatment can increase the colonization of *C. albicans* in the gastrointestinal tract and the presence of this fungus can hamper recovery of microbiota (Erb Downward et al., 2013). The different niches within the human body may provide different amounts of available iron due to conditions at these bodily sites, including oxygen availability and pH. This includes the low availability of iron during systemic infection, compared to the more iron replete environments of the oral cavity and gastrointestinal tract. Conditions of the gastrointestinal tract, including decreased oxygen availability and pH, may promote the ferrous iron form which is more soluble compared to its ferric, oxidized state and thus more bioavailable (Lambooij et al., 2017). Colonic microbiota may also influence the valence state of iron by the action of extracellular reductases (Cowart, 2002). Interestingly, although sufficient iron may be present in the gastrointestinal tract, more specifically the colonic lumen, mostly due to excess dietary iron, it may have limited availability to microbiota. This is due to dietary molecules such as polyphenol and phytate as well as metabolic by-products that sequester available iron (reviewed by Kortman et al., 2014).

Tissue infection may expose the host as well as pathogens to hypoxia at the site of infection due to decreased tissue perfusion, microvascular injury, or metabolic activities of pathogens and inflammatory cells (Nizet and Johnson, 2009; Chung et al., 2012). In addition, host proteins such as ferritin are unstable at acidic pH, therefore, acidification of environments that contain iron-binding proteins may release protein-bound iron, making it available for use by microbes such as *C. albicans* (Almeida et al., 2008). An example of this is the acidification of an environment through acid production (such as lactate) by bacteria such as *Staphylococcus aureus*, a frequent co-isolate with *C. albicans* from patients' lungs with cystic fibrosis (CF) as well as Streptococci (Bauernfeind et al., 1987; Friedman et al., 2006; Chotirmall et al., 2010). This would lead to the formation of the preferred ferrous state of iron and release of iron from host proteins such as transferrin. Co-habitation of *C. albicans* with these bacteria may enable *C. albicans* to obtain iron more easily through a synergistic relationship. Interestingly, an increase in iron and iron-related proteins is found in the lungs of CF patients (Hunter et al., 2013; Quinn et al., 2014). In addition, the low pH of the CF lung, in part due to inflammation, may promote the ferrous form (Tate et al., 2002; Tyrrell and Callaghan, 2016). The availability of ferrous iron correlates with disease severity on CF patient lungs (Hunter et al., 2013). This provides a unique environment for pathogens, as more iron is available, that may increase the severity of infection. In addition, the lysis of bacterial or fungal cells may release iron into the environment that can act as a source for co-inhabitants. This phenomenon has been reported between co-habitants where *Pseudomonas aeruginosa* induces the lysis of *S. aureus* for iron (Mashburn et al., 2005; Barnabie and Whiteley, 2015; Nguyen et al., 2015). *P. aeruginosa* is another frequent co-isolate of *C. albicans* in the CF lung and reports indicate an antagonistic interaction between the fungus and bacterium *in vitro*. This includes physical killing of *C. albicans* by *P. aeruginosa* as well as inhibition of metabolic activity and biofilm formation by bacterial cell wall components and secreted factors, including phenazines and quorum sensing molecules (Brand et al., 2008; Holcombe et al., 2010; Bandara et al., 2013). This killing of *C. albicans* cells may provide co-inhabitants with iron. In addition, *in vitro* studies indicate that siderophore production by *P. aeruginosa* suppresses growth of *C. albicans* through iron sequestration (Purschke et al., 2012; Trejo-Hernández et al., 2014). Interestingly, *C. albicans* has been shown to repress *P. aeruginosa* siderophore production *in vivo*, indirectly reducing the virulence of the bacterium (Lamont et al., 2002; Lopez-Medina et al., 2015).

*P. aeruginosa* has become rather famous for its nutrient acquisition strategies, including iron. For example, *P. aeruginosa* produces phenazines, redox active molecules that can reduce ferric iron and promote the growth of the bacterium through increasing iron availability (Wang et al., 2011). Interestingly, Briard et al. (2015) showed that sub-inhibitory concentrations of phenazines produced by *P. aeruginosa* can reduce ferric iron, making it more bio-available for its CF co-inhabitant *A. fumigatus*. However, at higher concentrations the phenazine 1-hydroxyphenazine may chelate iron. A similar result may be expected for *P. aeruginosa* in the presence of other fungi such as *C. albicans* (Briard et al., 2015). Although co-inhabitants of *C. albicans* may elicit environmental changes to promote nutrient acquisition, *C. albicans* itself is known to rapidly decrease the oxygen in its environment and even promote the growth of obligate anaerobic bacteria in oxic environments (Lambooij et al., 2017). Due to the differences in oxygen availability in different bodily sites, this may encourage more pronounced alterations in areas that are normally associated with available oxygen, such as the lungs and oral cavity compared to the gastrointestinal tract, which is usually associated with hypoxia. This is especially interesting since the ferrous iron form is promoted in the absence of oxygen. Thus, one can speculate that *C. albicans* may shift the occurrence of the ferrous iron form making it more available for co-inhabitants.

As mentioned above, *C. albicans* can utilize xenosiderophores from other co-inhabitants in a process known as “siderophore piracy” (Holzberg and Artis, 1983; Haas, 2003). The iron from these siderophores produced by co-inhabitants can be assimilated via both the reductive iron uptake system as well as the siderophore transporter Sit1/Arn1 (Heymann et al., 2002; Hu et al., 2002; Lee and Han, 2006). The Sit1/Arn1 siderophore uptake system can take up hydroxamate siderophores such as those produced by *Aspergillus fumigatus*, another co-isolate in CF, as well as other fungal-associated ferrichrome-type siderophores, whereas the reductive iron acquisition system can acquire iron from ferrioxamines, associated with bacterial origin (Heymann et al., 2002; Lesuisse et al., 2002). Therefore, *C. albicans* appears to possess dedicated uptake systems for ferrichrome siderophores (via Sit1/Arn1) and ferrioxamine siderophores (reductive iron uptake—Ftr1 dependent), whereas in *S. cerevisiae*, this distinction is less pronounced, as Sit1 can facilitate uptake of ferrioxamines as well as ferrichrome-type siderophores (Heymann et al., 2002). This change in specificity may be sequence specific, as CaSit1 shares only 46% identity with ScSit1, whereas CaSit1 also shares similarity to other proteins in *S. cerevisiae* that play roles in siderophore acquisition, such as Arn1 (51%), Arn2 (44%), and Enb1 (32%).

Except for *P. aeruginosa*, limited information focused on iron is available for the interaction of *C. albicans* with other pathogenic bacteria [i.e., *Streptococcus gordonii* binds to the ferritin receptor, Als3, of *C. albicans* (Liu and Filler, 2011)]. As iron can act as a driving force in population dynamics and due to its vital role in virulence, it is of considerable importance to focus research on the role of iron in polymicrobial infection. In addition, only speculative information is available in terms of the role of iron in the interaction of *C. albicans* with commensal organisms such as those found in the gastrointestinal tract and the role that it may play in *C. albicans* transition into pathogen from a commensal.

## Role of iron in treatment of *Candida albicans* infections

From the information presented in this review, it is apparent that dysregulation of iron in both the host and the pathogen can have consequences on the severity of infection. In the host, deviation from iron homeostasis, such as iron overload (haemochromatosis) or iron deprivation, can increase the host's susceptibility toward infection (Kumar and Choudhry, 2010). An example of this is the predisposition to fungal infection of leukemia patients due to disturbances in serum iron availability (Iglesias-Osma et al., 1995). In addition, iron can directly promote microbial growth (Cassat and Skaar, 2013), Zimmermann et al. (2010) reported an increase in enteric pathogens with iron supplementation. On the other side of the spectrum, Lu (2016) reported a high prevalence of oral candidosis for patients suffering from iron deficiency. This effect may be due to decreased immunity toward infection, including impaired cellular immunity, antibody response, and epithelial cell-alterations (Ishida and Johansen, 2014; Lu, 2016).

Iron overload in the host can be due to hereditary or dietary factors and the main therapy for this is through the administration of small molecule chelators (Meyer, 2006). These chelators can alter the development of inflammatory diseases, have antiviral properties and can also be used in the treatment of malignancies (Meyer, 2006; Lehmann et al., 2015). They also have potential as alternative treatment for multiple drug-resistant infections (Thompson et al., 2012). Iron overload in mice can hamper the protective Th1 response and ability to clear *C. albicans* infection (Mencacci et al., 1997; Ishida and Johansen, 2014). This effect can be rescued through treatment with the iron chelator deferoxamine. Although chelators show promise in treatment of some infections, they can also exacerbate certain types of infections such as mucormycosis and salmonellosis (Collins et al., 2002; Spellberg et al., 2005). This is because chelators such as deferoxamine have bacterial origins as siderophores and can be used by certain microorganisms, such as *Rhizopus* spp. in the case of mucormycosis, and *Salmonella typhimurium* in the case of salmonellosis (Perloth et al., 2007). However, newer generations or synthetic chelators may circumvent this problem. An example is the hydroxypyridone antimycotic, ciclopirox olamine, used for topical treatment of mucocutanous mycoses, which has been shown to exhibit strong antimicrobial properties toward *C. albicans* as well as *Cryptococcus* spp. through chelation of iron (Niewerth et al., 2003; Lee et al., 2005; Oliveira et al., 2010). Repurposing this drug toward drug-resistant bacterial species has also proven to be promising (Carlson-Banning et al., 2013). Strikingly, in contrast to fluconazole, no resistance toward the chelator, ciclopirox olamine, has been reported, indicating the importance of research into treatments that limit microbial growth through inhibition of micronutrient acquisition.

Several other strategies to limit iron acquisition by *C. albicans* have been investigated. The development of a monoclonal antibody, denoted C7, proved its potential as a therapeutic agent by its ability to induce iron starvation in *C. albicans*, resulting in strong antifungal properties (Brena et al., 2011). This effect on *C. albicans* is through the blocking of the reductive iron uptake pathway in the yeast. Administration of the glycoprotein lactoferrin can be a valid prospect for *C. albicans* treatment through not only sequestration of iron, but also direct toxicity and this led to the development of a mucoadhesive tablet containing lactoferrin for the treatment of oropharyngeal candidosis (Kuipers et al., 2002; Bai et al., 2006; Velliyagounder et al., 2015). Similar to this, administration of supraphysiological concentrations of transferrin limits microbial growth and show anti-*Candida* properties (Han, 2005, 2014a; Lin L. et al., 2014). Large iron chelator molecules such as transferrin and lactoferrin have reduced toxicity to the host compared to small molecule chelators. Interestingly, deletion of the *C. albicans MNN5* gene, encoding for a α-1,2-mannolsyltransferase, further increases susceptibility to lactoferrin possibly through the alteration of cell surface proteins that increase the toxicity of lactoferrin to *C. albicans* (Bai et al., 2006).

Research also suggests that using iron chelators as adjuncts during antifungal therapy may be beneficial, as iron deprivation can alter membrane fluidity and permeability, leading to increased susceptibility to antifungal agents (Prasad et al., 2006). The calcineurin pathway, involved in stress response in fungi, is supressed during iron deprivation, which leads to hypersensitivity to stresses, including membrane disturbances, such as those caused by antifungal drugs that target cell membrane homeostasis (Hameed et al., 2011). The hypersusceptibility to membrane perturbations as a result of iron starvation has previously also been reported for the bacterial pathogen *Mycobacterium tuberculosis* (Pal et al., 2015). Interestingly, iron deprivation in *C. albicans* can affect cell surface composition and daughter cells from iron-limited biofilms are more susceptible to amphotericin B than parent cells (Baillie and Doulgas, 1998). The tetracycline antibiotic, doxycycline, has been shown to also drastically increase the susceptibility of *C. albicans* to fluconazole, due to the ability of doxycycline to chelate iron and cause iron starvation in *C. albicans* (Fiori and Van Dijck, 2012). In addition, combination therapy of antifungal agents with lactoferrin also elicits a synergistic effect (Kuipers et al., 1999).

## Conclusions

Iron is an absolute requirement for most biological systems. The information discussed above describes the complex regulatory circuits for iron acquisition and homeostasis of both host and *C. albicans*. Considering the importance of iron in *C. albicans* growth and virulence, it can be expected that iron may play a significant role in the interaction of *C. albicans* with both commensal and pathogenic microorganisms in different niches within the human body. However, there is little information available regarding the role of iron during the interaction of *C. albicans* with co-inhabitants. Nutrient availability may alter population dynamics; therefore, iron availability and competition may affect disease outcome as well as *C. albicans* transition from commensalism to pathogenesis. Medical manipulation of iron availability in fighting infections, such as those caused by *C. albicans*, may prove to be a valuable addition to existing therapies. Given the frequent co-infection of *C. albicans* with other pathogens, as in the case of CF, manipulation of iron availability may also show potential in the treatment of these polymicrobial infections, however, further research is required.

## Author contributions

RF, OK, and BM compiled the information, co-wrote the manuscript and approved the final version submitted. JA provided scholarly input in placing the literature into context, edited the manuscript and approved the final version submitted. CP provided scholarly input in placing the literature into context, co-wrote the manuscript and approved the final version submitted.

### Conflict of interest statement

The authors declare that the research was conducted in the absence of any commercial or financial relationships that could be construed as a potential conflict of interest.
